# Supplementary material for: Resolving the full spectrum of human genome variation using Linked-Reads
Source: Genome Res. 2019 Apr;29(4):635–45. doi: 10.1101/gr.234443.118 (PMC6442396; doi:10.1101/gr.234443.118)
Supplement: Supplemental Material [file supp_gr.234443.118_Supplemental_longranger-2.2.zip › longranger-2.2.tar.bz2!/lr2.2-public/lib/stan/cmdstan/stan/doc/api/html/structstan_1_1math_1_1coupled__ode__system_3_01_f_00_01stan_1_1agrad_1_1var_00_01stan_1_1agrad_1_1var_01_4.html]

Stan: stan::math::coupled\_ode\_system< F, stan::agrad::var, stan::agrad::var > Struct Template Reference


|  |  |
| --- | --- |
|  | Stan  2.5.0  probability, sampling & optimization |


- Main Page
- Related Pages
- Namespaces
- Classes
- Files

- Class List
- Class Index
- Class Hierarchy
- Class Members

All Classes Namespaces Files Functions Variables Typedefs Enumerations Enumerator Friends Macros Pages

- stan
- math
- coupled\_ode\_system< F, stan::agrad::var, stan::agrad::var >

Public Member Functions |
Public Attributes |
List of all members

stan::math::coupled\_ode\_system< F, stan::agrad::var, stan::agrad::var > Struct Template Reference

The coupled ode system for unknown intial values and unknown parameters.
More...

`#include <coupled_ode_system.hpp>`

|  |  |
| --- | --- |
| Public Member Functions | |
|  | coupled\_ode\_system (const F &f, const std::vector< stan::agrad::var > &y0, const std::vector< stan::agrad::var > &theta, const std::vector< double > &x, const std::vector< int > &x\_int, std::ostream \*msgs) |
|  | Construct a coupled ODE system with unknown initial value and known parameters, given the base ODE system functor, the initial state of the base ODE, the parameters, data, and an output stream to which to write messages. More... |
|  | |
| void | operator() (const std::vector< double > &y, std::vector< double > &dy\_dt, double t) |
|  | Populates the derivative vector with derivatives of the coupled ODE system state with respect to time evaluated at the specified state and specified time. More... |
|  | |
| int | size () const |
|  | Returns the size of the coupled system. More... |
|  | |
| std::vector< double > | initial\_state () |
|  | Returns the initial state of the coupled system. More... |
|  | |
| std::vector< std::vector  < stan::agrad::var > > | decouple\_states (const std::vector< std::vector< double > > &y) |
|  | Return the basic ODE solutions given the specified coupled system solutions, including the partials versus the parameters encoded in the autodiff results. More... |
|  | |

|  |  |
| --- | --- |
| Public Attributes | |
| const F & | f\_ |
|  | |
| const std::vector  < stan::agrad::var > & | y0\_ |
|  | |
| std::vector< double > | y0\_dbl\_ |
|  | |
| const std::vector  < stan::agrad::var > & | theta\_ |
|  | |
| std::vector< double > | theta\_dbl\_ |
|  | |
| const std::vector< double > & | x\_ |
|  | |
| const std::vector< int > & | x\_int\_ |
|  | |
| const int | N\_ |
|  | |
| const int | M\_ |
|  | |
| const int | size\_ |
|  | |
| std::ostream \* | msgs\_ |
|  | |

## Detailed Description

### template<typename F> struct stan::math::coupled\_ode\_system< F, stan::agrad::var, stan::agrad::var >

The coupled ode system for unknown intial values and unknown parameters.

The coupled system has N + N \* (N + M) states, where N is size of the base ODE state vector and M is the number of parameters.

The first N states correspond to the base system's N states:

The next N+M states correspond to the sensitivities of the initial conditions, then to the parameters with respect to the to the first base system equation:

The next N+M states correspond to the sensitivities with respect to the second base system equation, etc.

If the original ode has a state vector of size N states and a parameter vector of size M, the coupled system has N + N \* (N

- M) states. (derivatives of each state with respect to each initial value and each theta)

Template Parameters
:   |  |  |
    | --- | --- |
    | F | the functor for the base ode system |

Definition at line 506 of file coupled\_ode\_system.hpp.

## Constructor & Destructor Documentation

template<typename F >

|  |  |  |  |
| --- | --- | --- | --- |
| stan::math::coupled\_ode\_system< F, stan::agrad::var, stan::agrad::var >::coupled\_ode\_system | ( | const F & | *f*, |
|  |  | const std::vector< stan::agrad::var > & | *y0*, |
|  |  | const std::vector< stan::agrad::var > & | *theta*, |
|  |  | const std::vector< double > & | *x*, |
|  |  | const std::vector< int > & | *x\_int*, |
|  |  | std::ostream \* | *msgs* |
|  | ) |  |  |

inline

Construct a coupled ODE system with unknown initial value and known parameters, given the base ODE system functor, the initial state of the base ODE, the parameters, data, and an output stream to which to write messages.

Parameters
:   |  |  |  |
    | --- | --- | --- |
    | [in] | f | the base ode system functor. |
    | [in] | y0 | the initial state of the base ode. |
    | [in] | theta | parameters of the base ode. |
    | [in] | x | real data. |
    | [in] | x\_int | integer data. |
    | [in,out] | msgs | output stream to which to print messages. |

Definition at line 532 of file coupled\_ode\_system.hpp.

## Member Function Documentation

template<typename F >

|  |  |  |  |  |  |
| --- | --- | --- | --- | --- | --- |
| std::vector<std::vector<stan::agrad::var> > stan::math::coupled\_ode\_system< F, stan::agrad::var, stan::agrad::var >::decouple\_states | ( | const std::vector< std::vector< double > > & | *y* | ) |  |

inline

Return the basic ODE solutions given the specified coupled system solutions, including the partials versus the parameters encoded in the autodiff results.

Parameters
:   |  |  |
    | --- | --- |
    | y | the vector of the coupled states after solving the ode |

Definition at line 665 of file coupled\_ode\_system.hpp.

template<typename F >

|  |  |  |  |  |
| --- | --- | --- | --- | --- |
| std::vector<double> stan::math::coupled\_ode\_system< F, stan::agrad::var, stan::agrad::var >::initial\_state | ( |  | ) |  |

inline

Returns the initial state of the coupled system.

Because the initial state is unknown, the coupled system incorporates the initial condition offset from zero as a parameter, and hence the return of this function is a vector of zeros.

Returns
:   the initial condition of the coupled system. This is a vector of length size() where all elements are 0.

Definition at line 653 of file coupled\_ode\_system.hpp.

template<typename F >

|  |  |  |  |
| --- | --- | --- | --- |
| void stan::math::coupled\_ode\_system< F, stan::agrad::var, stan::agrad::var >::operator() | ( | const std::vector< double > & | *y*, |
|  |  | std::vector< double > & | *dy\_dt*, |
|  |  | double | *t* |
|  | ) |  |  |

inline

Populates the derivative vector with derivatives of the coupled ODE system state with respect to time evaluated at the specified state and specified time.

Parameters
:   |  |  |  |
    | --- | --- | --- |
    | [in] | y | the current state of the coupled ode system, of size `size()`. |
    | [in,out] | dy\_dt | populate with the derivatives of the coupled system evaluated at the specified state and time. |
    | [in] | t | time. |

Exceptions
:   |  |  |
    | --- | --- |
    | exception | if the base system does not return a derivative vector of the same size as the state vector. |

Definition at line 570 of file coupled\_ode\_system.hpp.

template<typename F >

|  |  |  |  |  |
| --- | --- | --- | --- | --- |
| int stan::math::coupled\_ode\_system< F, stan::agrad::var, stan::agrad::var >::size | ( |  | ) | const |

inline

Returns the size of the coupled system.

Returns
:   size of the coupled system.

Definition at line 638 of file coupled\_ode\_system.hpp.

## Member Data Documentation

template<typename F >

const F& stan::math::coupled\_ode\_system< F, stan::agrad::var, stan::agrad::var >::f\_

Definition at line 507 of file coupled\_ode\_system.hpp.

template<typename F >

const int stan::math::coupled\_ode\_system< F, stan::agrad::var, stan::agrad::var >::M\_

Definition at line 515 of file coupled\_ode\_system.hpp.

template<typename F >

std::ostream\* stan::math::coupled\_ode\_system< F, stan::agrad::var, stan::agrad::var >::msgs\_

Definition at line 517 of file coupled\_ode\_system.hpp.

template<typename F >

const int stan::math::coupled\_ode\_system< F, stan::agrad::var, stan::agrad::var >::N\_

Definition at line 514 of file coupled\_ode\_system.hpp.

template<typename F >

const int stan::math::coupled\_ode\_system< F, stan::agrad::var, stan::agrad::var >::size\_

Definition at line 516 of file coupled\_ode\_system.hpp.

template<typename F >

const std::vector<stan::agrad::var>& stan::math::coupled\_ode\_system< F, stan::agrad::var, stan::agrad::var >::theta\_

Definition at line 510 of file coupled\_ode\_system.hpp.

template<typename F >

std::vector<double> stan::math::coupled\_ode\_system< F, stan::agrad::var, stan::agrad::var >::theta\_dbl\_

Definition at line 511 of file coupled\_ode\_system.hpp.

template<typename F >

const std::vector<double>& stan::math::coupled\_ode\_system< F, stan::agrad::var, stan::agrad::var >::x\_

Definition at line 512 of file coupled\_ode\_system.hpp.

template<typename F >

const std::vector<int>& stan::math::coupled\_ode\_system< F, stan::agrad::var, stan::agrad::var >::x\_int\_

Definition at line 513 of file coupled\_ode\_system.hpp.

template<typename F >

const std::vector<stan::agrad::var>& stan::math::coupled\_ode\_system< F, stan::agrad::var, stan::agrad::var >::y0\_

Definition at line 508 of file coupled\_ode\_system.hpp.

template<typename F >

std::vector<double> stan::math::coupled\_ode\_system< F, stan::agrad::var, stan::agrad::var >::y0\_dbl\_

Definition at line 509 of file coupled\_ode\_system.hpp.

---

The documentation for this struct was generated from the following file:

- src/stan/agrad/rev/ode/coupled\_ode\_system.hpp


---

[ Stan Home Page ]

*© 2011–2014,
Stan Development Team.*
